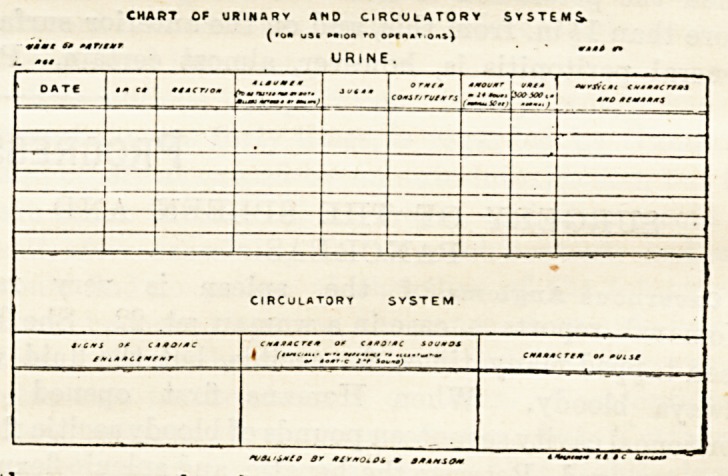# New Appliances and Things Medical

**Published:** 1898-03-26

**Authors:** 


### NEW APPLIANCES AND THINGS MEDICAL.

[We shall be glad to receive, at our Office, 28 & 29, Southampton Street, Strand, London, W.C., from the manufacturers, specimens of all new preparations and appliances which may be brought out from time to time.]

### "SIROL" ANTISEPTIC TOOTH PASTE.

(Gustav Hermanni, jun., 20, High Holborn, W.C.)

This tooth paste is contained in a collapsable tube, and a portion of the paste about the size of a pea is to be brushed on the teeth night and morning, and if possible after each meal. It is said to be absolutely uninjurious to the enamel. Sirol is a white paste of aromatic odour, and is very pleasant as a dentifrice. On analysis it was found to contain nothing that could injure the teeth; its ingredients are such as would serve as a neutralising agent to the acid secretions of the mouth, and its aromatic constituents act as an antiseptic. In these days of mal-dentition it should be remembered that the judicious use of some such acid-neutralising agent is frequently very efficacious in preserving the teeth from premature decay.

### HAY'S FOOD FOR INFANTS AND INVALIDS—HAY'S FRUMENTINE.

(Hygeian Flour Mills, Hull.)

Hay's Food is intended for the use of infants, invalids, and the aged, as being easily digestible and as containing not only all the necessary elements of nutrition found in human milk, but as supplying other necessary albumenoids. It is a cereal flour, partly cooked and malted, and makes a very palatable and nutritious food for invalids, and for children who are able to digest starchy foods, but it should not be given to infants under three months of age, as directed on the tin, considering that it contains over 60 per cent. of starchy material. Hay's Frumentine is another cereal flour more especially adapted for nursing mothers and adults suffering from defective mastication and constipation. It is a nutritious food, containing some bran of the meal in a finely divided condition, hence it may be useful and less irritating than brown bread to persons of sedentary habits suffering from constipation. Hay's Food and Frumentine are examples of well-prepared cereal foods.

### CHART OF URINARY AND CIRCULATORY SYSTEMS FOR USE PRIOR TO OPERATION.

(Reynolds and Branson, Leeds.)

It is every year becoming more generally recognised that the condition of the urinary organs and of the circulatory system of any patient on whom it is proposed to perform an operation should be thoroughly investigated. This is important, not only because the justifiability of the operation may, in more severe cases, largely hinge on the integrity of these organs, but because a complete knowledge of their condition may be essential to the anæsthetist. With the object of systematising the routine examination to which patients are subjected, Dr. A. Mackenzie, of Leeds, has arranged a chart on which the various essential points may be readily displayed. There can be no doubt that the use of this chart, and the habit of inquiring into all the details suggested by it, before performing any operation, will conduce to the safety of the patients.

## Figures and Tables

**Figure f1:**